# Cycloaliphatic Quaternary Ammonium Functionalized Poly(oxindole biphenyl) Based Anion-Exchange Membranes for Water Electrolysis: Stability and Performance

**DOI:** 10.3390/polym16010099

**Published:** 2023-12-28

**Authors:** Sara Gjoshi, Paraskevi Loukopoulou, Michaela Plevova, Jaromir Hnat, Karel Bouzek, Valadoula Deimede

**Affiliations:** 1 Department of Chemistry, University of Patras, GR-26504 Patras, Greece; saragjoshich@gmail.com (S.G.); evi_loukopoulou@outlook.com.gr (P.L.); 2 Department of Inorganic Technology, University of Chemistry and Technology, Prague, Technická 5, 16628 Prague, Czech Republic; michaela.plevova@vscht.cz (M.P.); jaromir.hnat@vscht.cz (J.H.); karel.bouzek@vscht.cz (K.B.)

**Keywords:** alkaline water electrolysis, AEMs, alkaline stability, piperidinium, pyrrolidinium, poly(oxindole biphenyl) backbone, ionic conductivity

## Abstract

Mechanically robust anion-exchange membranes (AEMs) with high conductivity and long-term alkali resistance are needed for water electrolysis application. In this work, aryl-ether free polyaromatics containing isatin moieties were prepared via super acid-catalyzed copolymerization, followed by functionalization with alkaline stable cyclic quaternary ammonium (QA) cationic groups, to afford high performance AEMs for application in water electrolysis. The incorporation of side functional cationic groups (pyrrolidinium and piperidinium) onto a polymer backbone via a flexible alkyl spacer aimed at conductivity and alkaline stability improvement. The effect of cation structure on the properties of prepared AEMs was thoroughly studied. Pyrrolidinium- and piperidinium-based AEMs showed similar electrolyte uptakes and no obvious phase separation, as revealed by SAXS and further supported by AFM and TEM data. In addition, these AEMs displayed high conductivity values (81. 5 and 120 mS cm^−1^ for pyrrolidinium- and piperidinium-based AEM, respectively, at 80 °C) and excellent alkaline stability after 1 month aging in 2M KOH at 80 °C. Especially, a pyrrolidinium-based AEM membrane preserved 87% of its initial conductivity value, while at the same time retaining its flexibility and mechanical robustness after storage in alkaline media (2M KOH) for 1 month at 80 °C. Based on ^1^H NMR data, the conductivity loss observed after the aging test is mainly related to the piperidinium degradation that took place, probably via ring-opening Hofmann elimination, alkyl spacer scission and nucleophilic substitution reactions as well. The synthesized AEMs were also tested in an alkaline water electrolysis cell. Piperidinium-based AEM showed superior performance compared to its pyrrolidinium analogue, owing to its higher conductivity as revealed by EIS data, further confirming the ex situ conductivity measurements.

## 1. Introduction

Hydrogen is expected to play a central role in the green transition, serving both as an energy storage medium and as a feedstock for the production of chemicals, materials and sustainable fuels [1,2]. Green hydrogen production from renewable energy sources using low-temperature water electrolysis has garnered much attention recently [3,4].

There are two low-temperature water electrolysis technologies, anion-exchange membrane water electrolysis (AEMWE) and proton-exchange membrane water electrolysis (PEMWE) that employ non-porous polymer-based ion-exchange membranes, allowing a more compact cell configuration (zero-gap configuration) [5,6,7,8,9,10]. Despite the high efficiency of PEMWEs, the use of expensive acid-tolerant stack hardware, as well as the use of platinum and iridium-based catalysts, strongly increases the cost of these devices. Consequently, AEMWE is considered a sustainable alternative, as it can employ abundant, low-cost platinum-free catalysts and less expensive cell components under alkaline operation [11]. In AEMWE, the polymeric membrane (AEM) conducts hydroxide ions; thus, pure-water-fed operation is possible and highly desired [12]. However, the typical operation with low-concentration alkaline electrolyte solution 1M is advantageous, since the alkaline solution facilitates hydroxide ion transport and significantly improves the catalyst performance. Thus, mechanically robust AEMs with high hydroxide conductivity and alkaline stability are needed to boost the durability and high performance of AEMWE technology [10,13].

Over the past years, many efforts have been devoted to enhancing conductivity, either by increasing the number of cationic groups, which inversely results in deterioration of mechanical strength due to very high ion-exchange capacity (IEC), or by building interconnected ionic channels via the design of well-organized polymer architectures. Thus, well-defined architectures such as comb-shaped, grafted, block and side-chain-type AEMs were proposed to facilitate hydrophilic/hydrophobic microphase separation, thus enabling fast ion conduction [14,15,16,17].

On the other hand, alkaline stability is largely dictated by the polymeric backbone, the fixed cationic groups and the precise position of cationic groups in the polymeric structure. Under alkaline conditions, polymeric backbones containing main-chain aryl-ether linkages such as polysulfones, poly ether ether ketones and poly(phenylene oxide)s were found to be liable to hydroxide attack, especially if triggered by the presence of electron-withdrawing groups [18]. This in turn results in a conductivity decrease and poor mechanical properties, leading to failure of the device. To tackle this problem, novel AEM chemistries based on ether-free backbones such as polyphenylenes, poly(arylene alkylene)s, polystyrenes and poly(perfluoro alkyl phenylene)s have been tested [19,20,21,22]. Regarding the selection of alkaline stable cations, although the commonly used quaternary ammonium (QA) groups are unstable under alkaline conditions via well-documented mechanisms such as Hoffman β-elimination and nucleophilic substitution, N-alicyclic quaternary ammonium (including piperidinium- and pyrrolidinium-based AEMs) possess much higher alkaline stability, due to their high steric hindrance and low ring strain [23]. Moreover, alkaline stability strongly depends on the precise position of cations in the polymer structure. Accordingly, the attachment of a long alkyl spacer between the polymer backbone and QA cations yield, in side-chain-type AEMs that possess excellent alkaline stability due to the spacer, increased hydrophobicity and steric hindrance around the cationic center [24,25].

Super acid-catalyzed polyhydroxyalkylation was used as a simple route for preparing aryl-ether free polyaromatics that can be easily functionalized with stable cationic groups to afford high performance AEMs [26,27,28,29,30,31,32]. Zhang et al. synthesized QA-tethered poly(isatin biphenylene) with an aryl-ether free backbone and the obtained AEMs exhibited high hydroxide conductivity and high alkaline stability in 2M NaOH at 80 °C for 1050 h [27]. The strategy of placing stable cationic groups on flexible spacer units tethered to the polymer backbone is beneficial, leading to highly conductive and alkaline-stable membranes, as well as increased AEMWE performance. For example, Cha et al. synthesized polycarbazole-based AEMs containing flexible alkyl side chains with trimethyl ammonium pendants that achieved a high performance of 3.5 A cm^−2^ at 1.9 V [33]. Chen et al. developed poly(fluorenyl-*co*-aryl piperidinium) (PFAP)-based AEMs that were also used in an anhydrous cathode AEMWE, demonstrating a very high performance of 7.68 A cm^−2^ at 2.0 V in 1M NaOH [34].

In light of these data, in this work, we propose a copolymerization strategy to produce side-chain-type heterocyclic QA-based poly(oxindole biphenylene)s via two steps. First, super-acid polyhydroxyalkylation of isatin, trifluoroacetophenone and biphenyl yielded in precursor poly(oxindole biphenylene)s, followed by the functionalization of the N-H group of isatin with bromopentyl-functionalized piperidinium or bromopentyl-functionalized pyrrolidinium to yield in the respective AEMs. Poly(oxindole biphenylene) was selected as backbone due to its exceptional alkaline stability associated with the high energy of the lowest unoccupied molecular orbital (LUMO) of deprotonated oxindole anions [27,32,35,36]. The incorporation of a long, hydrophobic pentyl spacer between the polymer backbone and alkaline-stable piperidinium/pyrrolidinium aimed at improving conductivity and alkaline stability. Specifically, the hydrophobic pentyl spacer is not only responsible for inducing micro-phase separation and thus promoting ionic conductivity, but it is also expected to increase the alkaline stability by increasing backbone hydrophobicity and steric hindrance around the cationic center. In addition, it can control the swelling, thus resulting in improved mechanical properties of the membranes.

Since the two AEMs had a similar ion-exchange capacity, the effect of cation structure on physicochemical properties, morphology, ion conductivity and alkaline stability was investigated. A direct comparison of the electrolysis performance of the two different hetero-cycloaliphatic QAs was conducted.

## 2. Materials and Methods

### 2.1. Materials

*N*-methyl pyrrolidine (98%, Alfa Aesar, Haverill, MA, USA), *N*-methyl piperidine (99%, Alfa Aesar, Haverill, MA, USA), 1,5 dibromopentane (98%, Alfa Aesar, Haverill, MA, USA), potassium carbonate (K_2_CO_3_, 99%, Merck, Rowe, NJ, USA), biphenyl (99%, Alfa Aesar, Haverill, MA, USA), 2,2,2-trifluoroacetophenone (98%, Alfa Aesar, Haverill, MA, USA), isatin (98%, Alfa Aesar, Haverill, MA, USA), trifluoroacetic acid (TFA, 99%, Alfa Aesar, Haverill, MA, USA), trifluoromethanesulfonic acid (TFSA, 99%, Fluorochem, Derbyshire, UK), potassium hydroxide (+85% pellets, Merck, Rowe, NJ, USA), acetonitrile, (99.5%, Scharlab, Quezon City, Philippines), dimethylsulfoxide (DMSO, Scharlab, Quezon City, Philippines), *N*-methyl pyrrolidone (NMP, Scharlab, Quezon City, Philippines) acetone (Scharlab, Quezon City, Philippines), diethyl ether (Sigma Aldrich, St. Louis, MO, USA), methanol (Sigma Aldrich, St. Louis, MO, USA), dichloromethane (DCM, Scharlab, Quezon City, Philippines), dmso-*d*_6_ (99.80 atom% Deutero, Kastellaun, Germany) were all used as received.

### 2.2. Preparation of 1-(5-Bromopentyl)-1-methylpyrrolidinium Bromide (BrPyr) and 1-(5-Bromopentyl)-1-methylpiperidinium Bromide (BrPip)

**Scheme 1:**
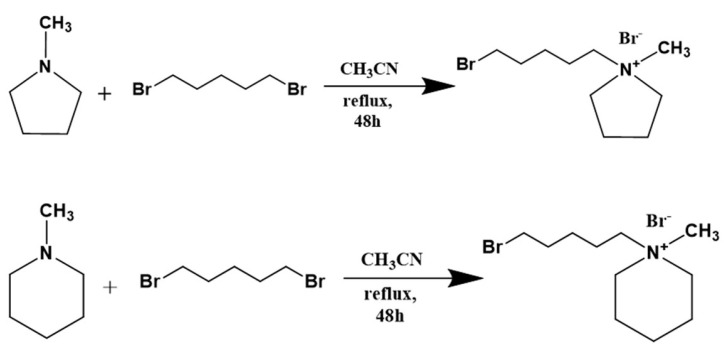
Synthesis of bromo-pentyl pyrrolidinium (BrPyr) and piperidinium analogue (BrPip).

The synthesis of 1-(5-bromopentyl)-1-methylpyrrolidinium bromide (BrPyr) was conducted according to the literature [27]. N-methyl pyrrolidine (4.3 mL, 40 mmol), 1,5 dibromopentane (54.2 mL, 400 mmol) and acetonitrile (220 mL) were added in a dry degassed 500 mL flask. The reaction mixture was heated under reflux (70–75 °C) for 48 h under argon atmosphere. The reaction mixture was evaporated under reduced pressure and the two-phase mixture obtained was separated. The oily phase was then precipitated in diethyl ether to yield the product. The product was dried under vacuum at 40 °C for 18 h. The reaction for the synthesis of BrPyr is shown in Scheme 1.

The chemical structure of BrPyr was characterized by ^1^H NMR as shown in Figure S1 (δ = 1.38–1.48 (m, 2H), δ = 1.70–1.80 (m, 2H), δ = 2.13 (s, 4H), δ = 2.95 (s, 3H), δ = 3.25–3.35 (m, 2H) and δ = 3.40–3.50 (m, 6H)).

The same procedure was followed for the synthesis of 1-(5-bromopentyl)-1-methylpiperidinium bromide (BrPip) (Scheme 1). The chemical structure was confirmed by ^1^H NMR as shown in Figure S2 ((δ = 1.4–1.48 (m, 2H), δ = 1.55–1.65 (m, 2H), δ = 1.70–1.80 (m, 2H), δ = 2.8 (s, 4H), δ = 2.85–2.9 (m, 2H), δ = 2.95 (s, 3H), δ = 3.2–3.3 (m, 6H) and δ = 3.40–3.45 (m, 2H)).

### 2.3. Synthesis of Poly(oxindole biphenyl-co-trifluoroacetophenone) Copolymers P(OBx-TFAP)

The synthesis of aromatic copolymers bearing isatin functionalities was carried out via superacid-catalyzed step-growth polymerization according to the literature, with minor modifications [37]. A typical example of polymer preparation is as follows. Isatin (1.43 g, 9.73 mmol), biphenyl (2 g, 12.97 mmol), 2,2,2-trifluoroacetophenone (1.13 g, 6.48 mmol), DCM (2.6 mL) and TFA (11.7 mL) were added into a dry degassed flask, cooled to 0–5 °C using an ice bath and stirred for 0.5 h. Subsequently, 13.4 mL of TFSA was slowly added into the mixture that was left at room temperature under vigorous stirring for 24 h until a highly viscous dark-green solution was obtained. Then, it was precipitated into a mixture of deionized water/methanol (2/1) and a white fibrous polymer was formed. The polymer was filtered off, washed with deionized water and finally dried overnight in vacuum oven at 60 °C and isolated in 85–95% yield. The synthesized copolymers are hereafter referred to as P(OBx-TFAP), where *x* denotes the molar percentage of oxindole-biphenyl segment (OB abbreviation) and TFAP corresponds to trifluoroacetophenone monomer abbreviation.

### 2.4. Synthesis of Pyrrolidinium-Functionalized Copolymers P(OB70pyr-TFAP) and Piperidinium-Functionalized Copolymers P(OB73pip-TFAP)

Both P(OB70pyr-TFAP) and P(OB73pip-TFAP) were synthesized following the same procedure. The preparation of P(OB70pyr-TFAP) is as follows. Accordingly, precursor copolymer P(OB70-TFAP) (1.00 g, 3.43 mmol) was fully dissolved in NMP (35 mL) at room temperature. Then, BrPyr (3.24 g, 10.3 mmol) and K_2_CO_3_ (0.95 g, 6.86 mmol) were added into portions to the above polymer solution. The reaction mixture was heated under reflux (70–75 °C) for 48 h under argon atmosphere and then poured into a large amount of acetone to obtain the product. The precipitate was washed with water and dried overnight in a vacuum oven at 60 °C.

### 2.5. Membrane Preparation

Cationic polymers in the as-synthesized Br^−^ form were dissolved in DMSO to obtain 5 wt% solution. The solution was filtered through filter paper and then cast onto a clean glass plate in an oven at 80 °C for 24 h. Transparent and flexible membranes were peeled off from the glass plate and dried in a vacuum oven at 80 °C for at least 20 h. The AEMs in the bromide form were ion-exchanged to the OH^−^ form by immersion in aqueous 1M KOH at room temperature for 3 days. During this time, the solution was replaced several times with fresh KOH to ensure full exchange. The membranes were thoroughly washed with deionized water and used for characterization.

### 2.6. Characterization

The proton nuclear magnetic resonance (^1^H NMR) spectra were recorded on an Advance DPX 400 MHz spectrometer (Bruker, Karlsruhe, Germany). The samples were dissolved in DMSO-*d*_6_ (or DMSO-*d*_6_/TFA), and the chemical shifts are reported relative to tetramethylsilane (TMS), which was used as internal standard. The intrinsic viscosity of P(OBx-TFAP) copolymers was determined at 25 °C, using an Ostwald viscometer with the copolymers dissolved in DMSO. The efflux time was measured three times at six or seven different concentrations of each sample. The reduced and inherent viscosities were calculated according to the following Equations (1) and (2):

(1) $$\eta_{inh}=\frac{\ln\left(\frac{t_{1}}{t_{2}}\right)}{c}$$

(2) $$\eta_{red}=\frac{\frac{t_{1}}{t_{2}}-1}{c}$$

where *t*_1_ is the efflux time for the polymer solution with concentration *c* and *t*_2_ is the efflux time for the blank sample. The intrinsic viscosity was determined by extrapolating both *η_inh_* and *η_red_* to zero concentration and calculating the average intersection with the *y*-axis.

Attenuated Total Reflection Fourier Transform Infra-Red (ATR-FTIR) spectra were conducted on a Platinum ATR spectrometer (Bruker).

Thermogravimetric analysis (TGA, Labsys TG, Setaram Instrumentation, Caluire, France) was carried out in the temperature range from room temperature to 800 °C under nitrogen atmosphere and a heating rate of 20 °C min^−1^.

Transmission Electron Microscopy (TEM) images were recorded using a JEM-2100 electron microscope (JEOL, Tokyo, Japan) at a working voltage of 120 kV. The specimen was prepared by casting an AEM thin film onto a Cu grid.

The Atomic Force Microscopy (AFM) measurements were performed using a Dimension Icon microscope (Bruker Co., USA) in Tapping Mode.

Small-Angle X-Ray Scattering (SAXS) measurements were conducted via a Xeuss 3.0 (Xenocs, Grenoble, France). Data were collected using incident X-rays with λ = 1.542 Å for scattering vectors (q) between 0.12 and 7.5 nm^−1^. The magnitude of the scattering vector was *q* = (4π/λ)sin θ, where 2θ is the scattering angle. All measurements were conducted at 25 °C.

### 2.7. Ion-Exchange Capacity (IEC) of the AEMs

The IEC of the AEM membranes in the bromide form was determined by Mohr’s titration. The membranes were dried at 80 °C under vacuum for 24 h and weighed to obtain their dry weights. Then, they were immersed in 25 mL 1M aq. NaNO_3_ solution for 3 days. The solution was titrated with a 0.01M aq. AgNO_3_ solution, using an aq. K_2_CrO_4_ solution (5%) as the indicator. The *IEC* was calculated according to Equation (3):

(3) $${IEC}_{{Br}^{-}}=\frac{C_{{AgNO}_{3}\;\cdot \;}V_{{AgNO}_{3}}}{m_{dry}}$$

where m_dry_ (g) is the mass of the dry membranes and the V_AgNO3_ (mL) represents the consumption volume of AgNO_3_.

For evaluation of the IEC prior and after the alkaline water electrolysis test, organic elemental analysis was used. The content of the nitrogen in the sample of the membrane was evaluated using Elementar vario El Cube analyser (Elementar, Langenselbold, Germany). Theoretical IEC was determined from the known mass of the dry sample and corresponding content of the nitrogen.

### 2.8. Water, Electrolyte Uptake and Swelling Ratio

Prior to water or electrolyte uptake and swelling measurements, the membrane samples were dried under vacuum at 80 °C for 24 h. After weighing, the membranes were immersed in water or 2M aqueous KOH solution at 20, 40, 60 and 80 °C for 24 h. The membranes were then quickly wiped to remove surface water or electrolyte and weighed again. The water uptake, *WU*, or electrolyte uptake, *EU* (i.e., the sum contributions from KOH and water), which is the difference in the weights before (*m_dry_*) and after soaking the membranes in water or electrolyte solution (*m_wet_*), was calculated according to Equation (4):

(4) $$WU\;or\;EU=\frac{m_{wet}-m_{dry}}{m_{dry}}\times 100\;\%$$

The swelling ratios *SR_a_* and *SR_t_* were determined based on the measured area (*a*) and thickness (*t*) before (dry dimensions) and after immersion (wet dimensions) in water or 2M KOH electrolyte solution at 20, 40, 60 and 80 °C for 24 h and calculated according to the following Equations (5) and (6):

(5) $${SR}_{a}=\frac{a_{wet}-a_{dry}}{a_{dry}}\times 100\;\%$$

(6) $${SR}_{t}=\frac{t_{wet}-t_{dry}}{t_{dry}}\times 100\;\%$$

where *a_dry_* and *a_wet_* are the areas (length × width) of the dry and wet membranes, and *t_dry_, t_wet_* are the thicknesses of the dry and wet membranes, respectively.

### 2.9. Ionic Conductivity

The through-plane ionic conductivity of the membranes was measured at 20, 40, 60, 80 °C by ac impedance spectroscopy. The measurements were carried out using a home-made, two compartment PTFE cell on AUTOLAB electrochemical workstation (PGSTAT 302N) equipped with a frequency response analyzer ranging from 10 Hz to 1000 kHz. The membranes were sandwiched between two perforated nickel-plate electrodes of active area 4 cm^2^ and the two compartments of the cell were filled with 2M KOH solution. Before conductivity measurements, the samples were equilibrated in the respective doping solution at 80 °C for 18 h. The ionic conductivity was calculated according to Equation (7), where *l* is the thickness of the membrane, *A* is the active area of the membrane and *R* is the ohmic resistance between the electrodes (taken as the intercept with the real axis of the Nyquist plot).

(7) $$\sigma =\frac{l}{A\times R\;}$$

For each membrane, two samples were prepared and measured, and the average value of the two measurements was reported.

Evaluation of the ionic conductivity prior and after the alkaline water electrolysis test was performed in poly(methyl methacrylate) cell filled with mercury. The membrane sample was squeezed between two poly(methyl methacrylate) parts with a circular active area of 15.7 mm^2^, which were in the next step flooded with mercury. Pt wires were immersed in the mercury, which ensured electron contact with the membrane surface and prevented the carbonization of the membrane due to the atmospheric CO_2_. Electrochemical impedance spectroscopy (EIS) was used to evaluate resistance of the cell. Measurement was conducted at room temperature (23 °C) using LCR bridge Hameg 8118. EIS was measured in the frequency range from 200 kHz to 20 Hz with maximal amplitude of the perturbing signal 50 mV. Ohmic resistance was evaluated from the Nyquist plot. Equation (7) was used to evaluate ionic conductivity.

### 2.10. Alkaline Stability

The membranes were stored in sealed PTFE vials containing 2M aqueous KOH solutions at 80 °C for up to one month to assess their alkaline stability. The changes in ionic conductivity were recorded and the structural variation before and after alkaline stability test were also studied by ^1^H NMR, ATR-FTIR and TGA.

### 2.11. Alkaline Water Electrolysis Testing

The performance of the prepared AEMs was tested in a lab-scale single-cell alkaline water electrolyzer. Multi Autolab M204 potentiostat/galvanostat equipped with 10 A current booster and FRA32M module allowing use of EIS measurement was used to perform membrane alkaline water electrolysis experiments. Control software NOVA 2.14 was used to set and proceed with the experimental procedure and collect data. Ni foam electrodes of geometrical area 4 cm^2^ were used as both anode and cathode, respectively. Prior to measurements, membranes were immersed in 1M KOH solution at 23 °C (room temperature) for three days, at which time the solution was changed every 24 h. Membranes and electrodes were assembled with PTFE gaskets chosen to match the assembly, and 1M KOH liquid electrolyte was used as a circulating medium. Short-term performance in the form of the load curves was measured in potentiostatic regime in the cell voltage range from 1.5 to 2.0 V with step 50 mV at temperatures 50, 60 and 70 °C. The value of the current passing through the cell at corresponding cell voltage was recorded for 60 s. Average value of the current regime was addressed to the particular cell voltage. Three load curves were measured and averaged. EIS was used in potentiostatic regime. The maximal amplitude of the perturbing signal was 10 mV. EIS spectra were measured in the frequency range from 65 kHz to 0.1 Hz at cell voltages between 1.5 to 1.8 V. Equivalent electrical circuit (Figure S10) was used to evaluate EIS spectra. Stability curves were recorded under the same conditions as load curves measurements and at a current density of 250 mA cm^−2^ for 50 h. Cell voltage value was recorded each 60 s. Each 50th recorded cell voltage value is shown in the stability test figures to limit the number of points. No corrections were applied on the membrane alkaline water electrolysis or EIS data, except for involving the active surface of the membrane to recalculate measured current (A) to current density (mA cm^−2^) and ohmic resistance (Ω) to area ohmic resistance (Ω cm^2^).

## 3. Results and Discussion

### 3.1. Synthesis and Characterization of Pyrrolidinium-Functionalized Copolymers P(OB70pyr-TFAP) and Piperidinium-Functionalized Copolymers P(OB73pip-TFAP)

**Scheme 2:**
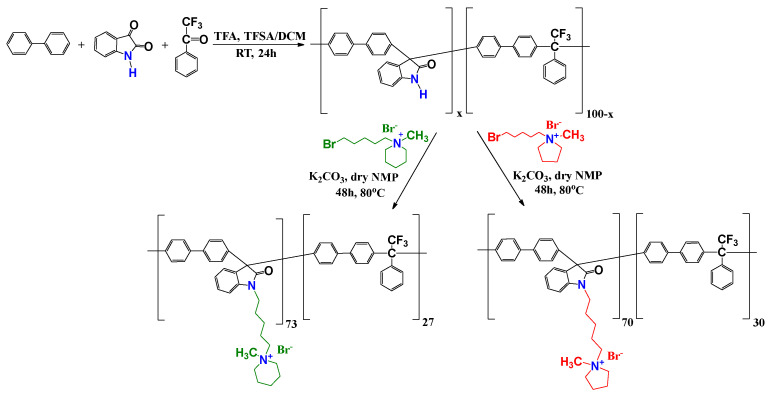
Synthesis pathway to poly(oxindole biphenyl) copolymers functionalized with long flexible cationic groups.

The development of high-performance AEMs for use in alkaline water electrolyzers is challenging, as it strongly depends on many critical factors, including chemical stability, ionic conductivity and mechanical robustness. Thus, to address these issues, the attachment of long side alkyl chains with alkali-resistant cationic pendants onto a mechanically stable, alkali-resistant polymeric backbone is proposed as a strategy. The introduction of long hydrophobic alkyl chains is expected to induce hydrophilic–hydrophobic phase separation and the formation of continuous ionic pathways, thus enhancing the ionic conductivity and improving the chemical stability as well. In addition, this hydrophobic alkyl spacer can regulate the water absorption, thus avoiding extremely high swelling. As shown in Scheme 2, two AEMs abbreviated as P(OB70pyr-TFAP) and P(OB73pip-TFAP) bearing different cationic side chains were synthesized in two steps, including the preparation of precursor copolymers containing isatin units in the main chain, followed by functionalization of the N-H group of isatin via the introduction of long side aliphatic chains containing terminal alkali-resistant piperidinium or pyrrolidinium groups.

First, the copolymerization of isatin and trifluoroacetophenone monomers with biphenyl in the presence of TFSA and TFA via the Friedel–Crafts polyhydroxyalkylation reaction yielded in ether free, high-molecular-weight precursor copolymers. Trifluoroacetophenone was selected as a comonomer to control the IEC of the resulting copolymers. To make a reasonable comparison, the molar ratio of isatin/trifluoroacetophenone was adjusted (73/27 and 70/30 for piperidinium- and pyrrolidium-based AEMs, respectively) to ensure that both AEMs had a similar IEC.

It should be mentioned that this reaction is a non-stoichiometric condensation, and a small molar excess of ketones monomers relative to biphenyl is needed to increase the polymerization rate and obtain high-molecular-weight polymers [38,39]. Indeed, the aforementioned copolymers were prepared using an excess of 25%. These copolymers had already been synthesized with different compositions for gas separation applications [37]. Interestingly, a stoichiometric ratio between ketones and biphenyl monomer (1/1) was used, while TFSA was the only catalyst of the reaction.

**Figure 1:**
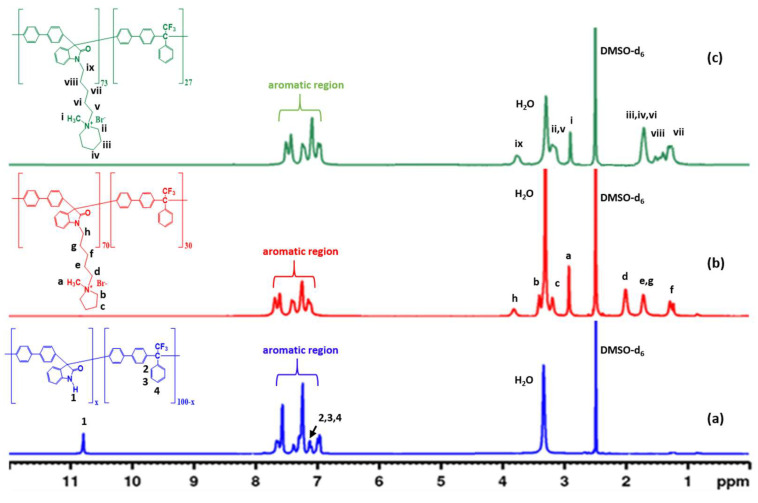
^1^H NMR spectra of (**a**) P(OBx-TFAP) copolymer, (**b**,**c**) the respective pyrrolidinium- and piperidinium-based copolymers in DMSO-d6.

The chemical structure of the P(OBx-TFAP) precursor copolymer was characterized by ^1^H NMR spectroscopy. As shown in Figure 1a, the N-H proton (**1**) in the isatin segment appeared at 10.80 ppm, while the signals for aromatic protons were observed at 6.95–7.70 ppm. The signal at 7.15 ppm could be attributed to the protons of phenyl group (**2**–**4**) in 2,2,2-trifluoroacetophenone moieties. The molar percentage of comonomers was calculated by comparing the integral areas of the N-H proton of isatin with the aromatic protons **2**–**4**. The calculated copolymer composition values agree well with the theoretical feed ratio, indicating the high reactivity of the monomers. The prepared copolymers are soluble in aprotic solvents like DMSO, NMP, *N*,*N*-dimethylacetamide (DMA) and *N*,*N*-dimethylformamide (DMF) but insoluble in common organic solvents (chloroform, tetrahydrofuran). Size exclusion chromatography measurements for the estimation of the molecular weights could not be performed due to its poor solubility in CHCl_3_. Thus, intrinsic viscosity [η] was measured in DMSO at 25 °C and found to be of 0.6 dL g^−1^, a value that is comparable with other aryl-ether free polyaromatics containing isatin groups synthesized via superacid-catalyzed polycondensation [29].

**Figure 2:**
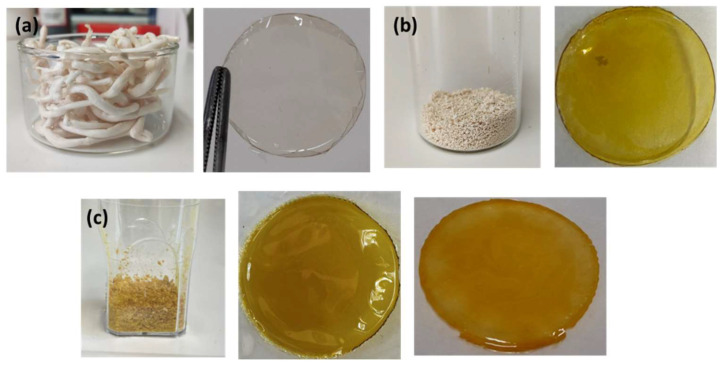
Photographs of flexible and transparent membranes prepared from (**a**) P(OBx-TFAP), (**b**) P(OB70pyr-TFAP) and (**c**) P(OB73pip-TFAP).

Copolymers P(OBx-TFAP) form mechanically flexible and robust transparent films upon casting from DMSO solutions, as evidenced in Figure 2. In the second step, functionalization of isatin via the introduction of long side alkali-resistant piperidinium or pyrrolidinium groups resulted in the respective AEM analogues. The cationic functionalized copolymers were synthesized via N-alkylation of isatin with BrPyr and BrPip, respectively. Specifically, the neutral copolymers were first dissolved in DMSO, and then anhydrous K_2_CO_3_ was added in portions to deprotonate the N-H proton of isatin, followed by partial addition of an excess of BrPyr or BrPip to depress cross-linking reactions.

The chemical structures of the prepared pyrrolidinium- and piperidinium-based copolymers were confirmed by ^1^H NMR spectroscopy using DMSO-d_6_ as solvent. The comparison of the ^1^H NMR spectra before and after functionalization confirmed the 100% successful functionalization of the precursor with cationic moieties in both cases (Figure 1b and Figure 1c, respectively), due to the absence of the peak at 10.80 ppm (**1**) which corresponds to the N-H proton in the isatin segment of the precursor copolymer (Figure 1a). Moreover, the integration ratio of α-methyl groups of cations to aromatic protons **2**–**4** is approximate to the theoretical value. Specifically, by taking the spectrum of P(OB73pip-TFAP) as an example, new signals from the piperidinium cation appeared between 1.15 and 1.65 ppm. In particular, the signal for the methylene protons ix adjacent to nitrogen of isatin were observed at 3.50 ppm, while the α-protons of piperidinium corresponding to methylene protons ii and v were observed at 3.20 ppm and the methyl protons i at 2.90 ppm, respectively. The methylene protons of the alkyl chain (vi, vii, viii) were observed in the region 1.15–1.65 ppm. Similarly, the signal at 1.65 ppm is ascribed to the -CH_2_- protons of the piperidinium ring (iii and iv).

To further confirm the successful incorporation of the cycloaliphatic QA groups, ATR spectroscopy was performed. The spectra of the synthesized precursor copolymer and the corresponding cationic analogues are depicted in Figure S3 for comparison reasons. In the spectrum of the precursor, the broad peak located at 3390 cm^−1^ is assigned to the N-H stretching of the isatin group, while the characteristic band at 1710 cm^−1^ corresponds to the C=O stretching of the amide bond [27,32]. After cationic functionalization, the broad peak observed at 3400 cm^−1^ was attributed to the characteristic stretching vibration of -OH groups of water molecules due to the hydrophilicity of cationic groups [40]. Two new absorption peaks arose at 2925 and 2860 cm^−1^ that are assigned to the stretching vibrations of methyl and methylene groups of alkyl spacer, respectively [41]. Therefore, these results in combination with the new peak observed at 1351 cm^−1^ corresponding to the stretching vibration of C-N^+^, confirm the successful incorporation of long side chain quaternized groups onto the polymeric backbone. The prepared cationic copolymers are soluble in DMSO and NMP but insoluble in common organic solvents (chloroform, tetrahydrofuran). The resulting membranes were transparent, mechanically stable and flexible, as shown in Figure 2b,c.

### 3.2. Thermal Stability

**Figure 3:**
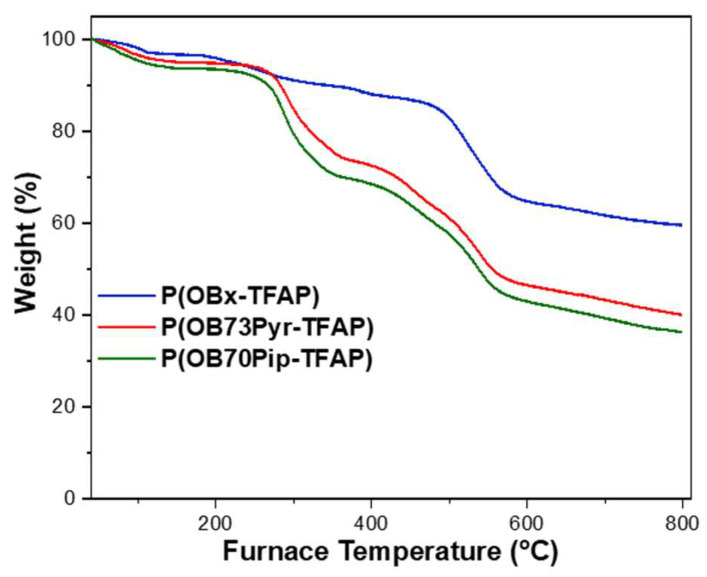
TGA curves of precursor and the two AEMs in bromide form.

The thermal stability of the precursor and the two AEMs in bromide form has been investigated by TGA analysis under N_2_ with a heating rate of 10 °C min^−1^, as depicted in Figure 3. A three-step weight loss was observed for both AEMs, while the precursor copolymer has superior thermal stability than the corresponding AEMs. An initial weight loss observed below 200 °C is related to the desorption of residual water and DMSO in the membranes. In the second step, decomposition of piperidinium/pyrrolidinium cationic groups was observed between 245 °C and 370 °C, in consistency with other reported piperidinium-based AEMs [29,31,42]. The third thermal decomposition, occurring between 380 °C and 560 °C, corresponds to the degradation of the polymeric backbone [37]. These results indicate that both AEMs exhibit sufficient thermal stability to be used in AEMWEs generally operating below 100 °C.

### 3.3. Morphology

The high conductivity of the AEMs is usually associated with an optimized nanomorphology of the membrane via the formation of well-developed percolating hydrophilic phase domains that are necessary for fast ion transport [26]. The flexibility and mobility of cationic chains significantly affect self-assembly and thus play an important role in the construction of ionic channels.

**Figure 4:**
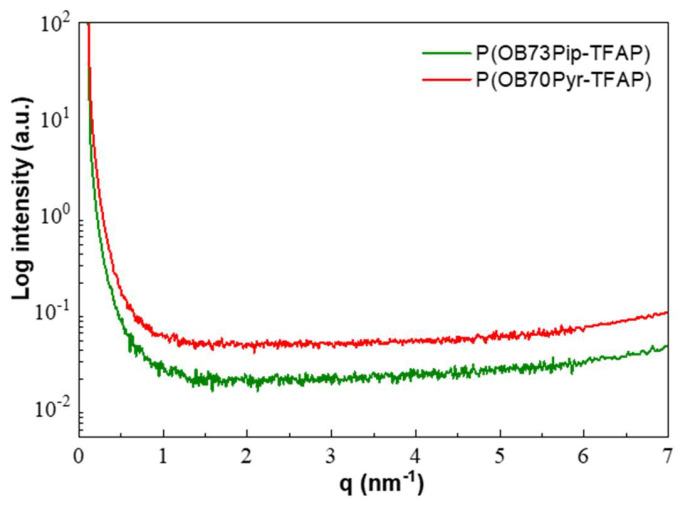
SAXS profiles of AEMs in the bromide form.

Therefore, SAXS and AFM measurements were performed to study any ionic clustering and phase separation of the AEMs. The characteristic separation length between ion-rich domains can be observed in terms of the values of q corresponding to the so-called ionomeric peak [26]. As shown in Figure 4, although there is a flexible *n*-pentyl spacer between the cations and rigid backbone which could potentially induce hydrophilic/hydrophobic separation, no ionomer peak was observed for both samples, suggesting that no obvious phase separation occurred. This is probably attributed to the insufficient ion aggregation due to the restricted mobility of cationic groups. In addition, the high polymeric backbone stiffness can prevent efficient ion clustering [25,27]. The same behavior was also reported for other side-chain-type AEMs with similar backbones [15,27,43]. AFM and TEM images further confirm that there is no distinct phase separation, as depicted in Figure S4. In the TEM images (Figure S4b,d), where the dark and the bright regions represent hydrophilic (ionic clusters) and hydrophobic domains, respectively, the size of dark domains is small and scattered and therefore cannot form a continuous ion transport channel. This is probably due to the insufficient phase separation, as ionic clusters are evenly dispersed in the polymer matrix and isolated from each other.

### 3.4. Water and Electrolyte Uptake and Swelling

Although AEMWEs typically operate with an electrolyte solution (i.e., 1M aqueous KOH), AEM properties such as water uptake, water swelling and conductivity are usually evaluated with pure water, because the research has been mainly focused on fuel cell applications. The present new AEMs were studied regarding both water and electrolyte uptake behavior. The IEC, WU, EU and SR at 20 and 80 °C of the prepared AEMs are listed in Table 1.

As shown in Table 1, the theoretical IEC values for pyrrolidinium and piperidinium AEMs calculated based on ^1^H NMR data are similar (1.54 mmol g^−1^ and 1.55 mmol g^−1^, respectively). The IEC experimental values determined by titration were 1.28 for the former and 1.34 mmolg^−1^ for the latter. This discrepancy is commonly observed for AEMs, as not all Br^−^ groups are accessible for ion exchange [25].

Water is necessary for the dissociation of OH^−^ ions and the construction of continuous ion-conducting channels for fast ion transfer across the membrane. The water uptake and swelling behavior were studied as a function of temperature (Figure S5 and Table 1). As expected, both membranes exhibited an increased water uptake and swelling ratio with increasing temperatures. P(OB70pyr-TFAP) displayed a moderate water uptake (24%), while P(OB73pip-TFAP) reached a 100% higher water uptake (48%) at 80 °C. In addition, both area swelling (28%) and thickness swelling values (29%) for P(OB73pip-TFAP) are higher than those of P(OB70pyr-TFAP) at 80 °C (11% and 14%, respectively), probably owing to the larger piperidine moiety facilitating the formation of bigger ionic clusters [43] and its slightly higher IEC. These results are in accordance with those reported in the literature [27,43,44,45]. Both membranes exhibit low swelling values attributed to the presence of flexible hydrophobic alkyl chains, which increase the volume fraction of hydrophobic domains, thereby restricting swelling and thus ensuring the good mechanical properties for application in alkaline water electrolyzers.

**Figure 5:**
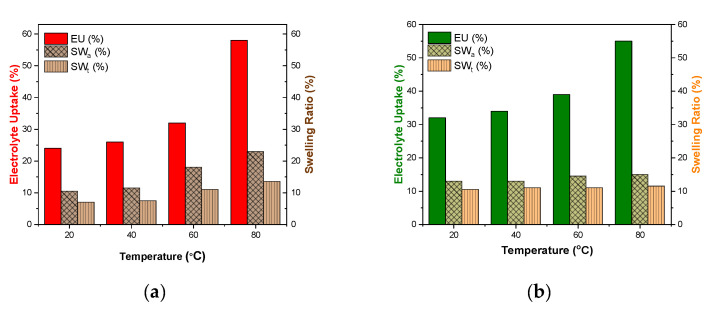
Electrolyte uptake and swelling behavior of (**a**) P(OB70pyr-TFAP) and (**b**) P(OB73pip-TFAP) in 2M KOH solution as a function of temperature.

Furthermore, an AEMs ability to be doped with dilute aqueous KOH electrolyte solution is a prerequisite to obtain high ionic conductivity in AEMWE. In Figure 5, the EU of the AEMs membranes after doping in 2M KOH solution is studied as a function of temperature. The same trend was also observed for electrolyte uptake, meaning that at higher temperatures, higher EU were reached. Both P(OB70pyr-TFAP) and P(OB73pip-TFAP) membranes exhibited similar EU values at 80 °C, 58 and 55%, respectively. These high electrolyte uptake values can be probably attributed to ionic interactions between C=O of isatin and cations or K^+^ [36]. Regarding swelling (Figure 5), P(OB73pip-TFAP) showed a lower swelling area value of 15% compared to that of P(OB70pyr-TFAP) at 80 °C (23%). In addition, the former showed good swelling resistance against a temperature increase from 20 to 80 °C, as almost no change in swelling thickness was observed. These results support that the prepared AEMs can be used in AEMWE fed with 2M electrolyte solutions.

### 3.5. Ionic Conductivity

**Figure 6:**
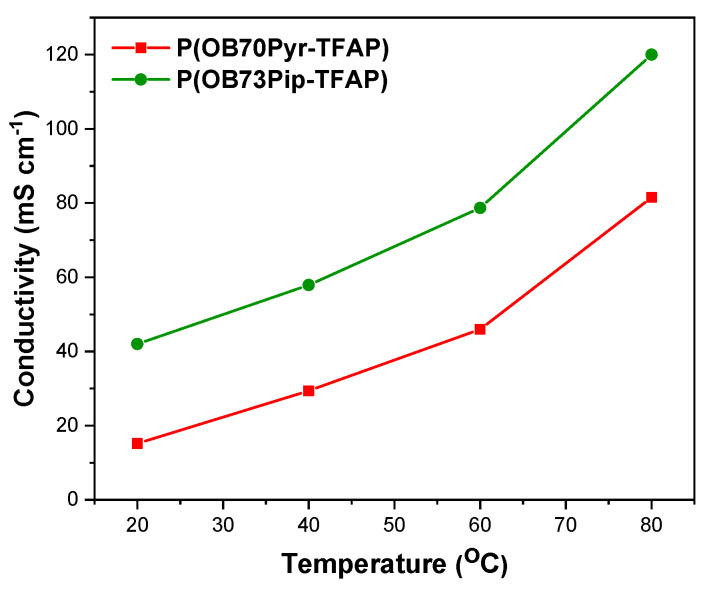
Ionic conductivity of AEMs in 2M KOH solution as a function of temperature.

Ionic conductivity is closely related to the performance of AEMWE. As shown in Figure 6, the conductivity was measured in 2M KOH solution in the temperature range of 20–80 °C. As expected, the conductivity increased with increasing temperature for both AEMs, due to the increased electrolyte absorption and ion mobility. Notably, the P(OB73pip-TFAP) membrane exhibited higher ionic conductivity values than the P(OB70pyr-TFAP) membrane in the whole temperature range. In particular, the former exhibited a value of 120 mS cm^−1^, while the latter exhibited a value of 81.5 mS cm^−1^ at 80 °C. It should be mentioned that piperidinium-based AEM showed higher electrolyte uptakes at 20, 40 and 60 °C, consequently resulted in higher conductivity than its pyrrolidinium analogue. However, at 80 °C, despite both AEMs displaying similar electrolyte uptakes, the conductivity of piperidinium was still significantly higher than pyrrolidinium. In addition, it is evident from Figure 6 that the slope has changed at 60 °C, indicating that the conductivity mechanism is also altered. This can be related to some morphological rearrangements that took place from 60 to 80 °C that probably led to an increased mobility of piperidinium chains. It should be noted that direct comparison of the conductivity data of the present work with previously reported data on other side alkyl poly(oxindole biphenylene) based AEMs cannot be conducted as most are recorded in pure water. However, very recently, polybenzimidazole tethered with *N*,*N*-dimethylpiperidinium cations was reported for use in dilute aqueous KOH-fed electrolyzers. The conductivity of the prepared membranes reached values ranging from 19 to 38 mS cm^−1^ in 2M KOH at 80 °C [46].

### 3.6. Alkaline Stability

**Figure 7:**
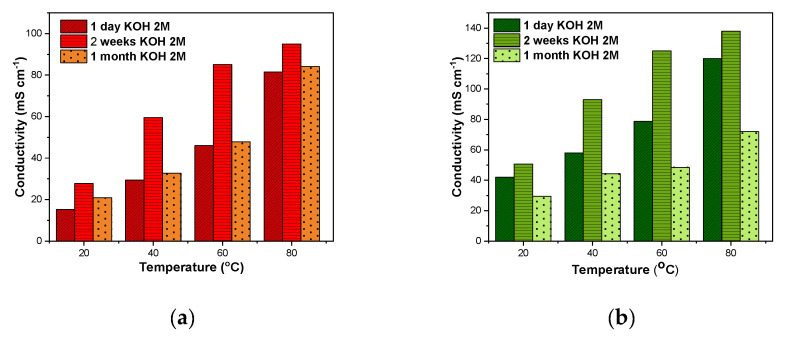
Alkaline stability of (**a**) pyrrolidinium- and (**b**) piperidinium-based AEMs in 2M KOH at 80 °C after 1 day, 2 weeks and 1 month.

The long-term AEMWE performance is strongly dependent on the chemical stability of AEMs. As the aryl-ether free poly(oxindole biphenylene) backbone is known to be highly alkaline-stable in high-concentrated KOH solution (8M) [35], we focused on the alkaline stability of the cations. The synthesized AEMs were immersed in 2M aq. KOH for 1 month at 80 °C in order to study their alkaline stability. The changes in conductivity were recorded as shown in Figure 7.

**Figure 8:**
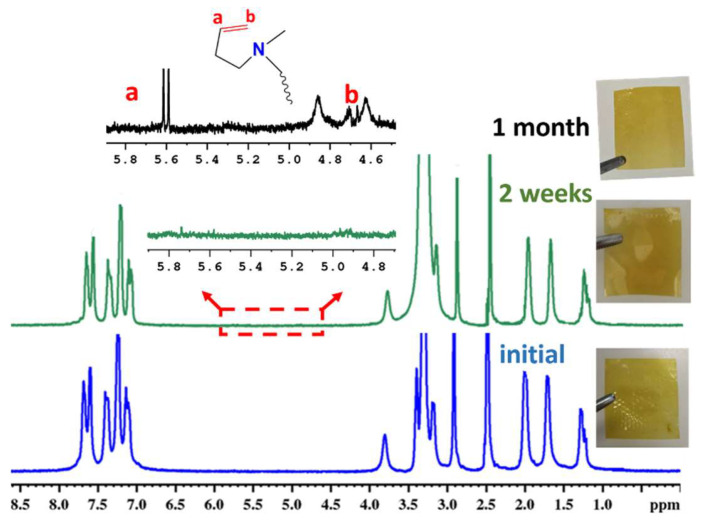
^1^H NMR spectra of pyrrolidinium-based AEM before and after storage in 2M KOH for 1 month at 80 °C.

It is evident that the conductivity of both membranes was increased for the first 2 weeks’ storage in 2M KOH, probably related with the not-100%-efficient anion exchange to OH^−^ ions. Another explanation could be that the strong plasticization induced by the electrolyte at elevated temperatures (80 °C) enables the absorption of higher amounts of electrolyte, thus promoting conductivity [45]. In addition, despite pyrrolidinium-based AEM showing a very small conductivity loss for the next 2 weeks (13%), the conductivity after 1-month storage remained almost unchanged compared to the initial value, suggesting its excellent chemical stability. On the contrary, piperidinium-based AEM showed a significant conductivity decline, maintaining 52% of its initial conductivity. The excellent alkaline resistance of the former could be ascribed to the stability of the cation and possibly to morphological changes during the alkali treatment [20]. The increased alkaline resistance of pyrrolidinium compared to piperidinium was also reported for other AEMs after alkaline treatment at 60 and 80 °C [44,47]. In order to investigate the reason for the conductivity loss, the aged samples were characterized by ^1^H NMR. ^1^H NMR spectra of P(OB70pyr-TFAP) before and after the alkali treatment are displayed in Figure 8. The solubility of the P(OB70pyr-TFAP) membrane was reduced with increasing storage time in the alkaline solution, probably due to cross-linking reactions [27]. Accordingly, the membrane was only partially soluble in DMSO-d_6_ after 1 month of alkaline treatment. For this reason, the data cannot be used for quantitative analysis of the degradation products. Although no new peaks were observed after 2 weeks, some new, very small peaks emerged in the region 4.3–5.6 ppm after one-month aging, corresponding to vinylic protons originating from the degradation of pyrrolidinium via ring-opening Hofmann β-elimination. [23,27,48].

**Figure 9:**
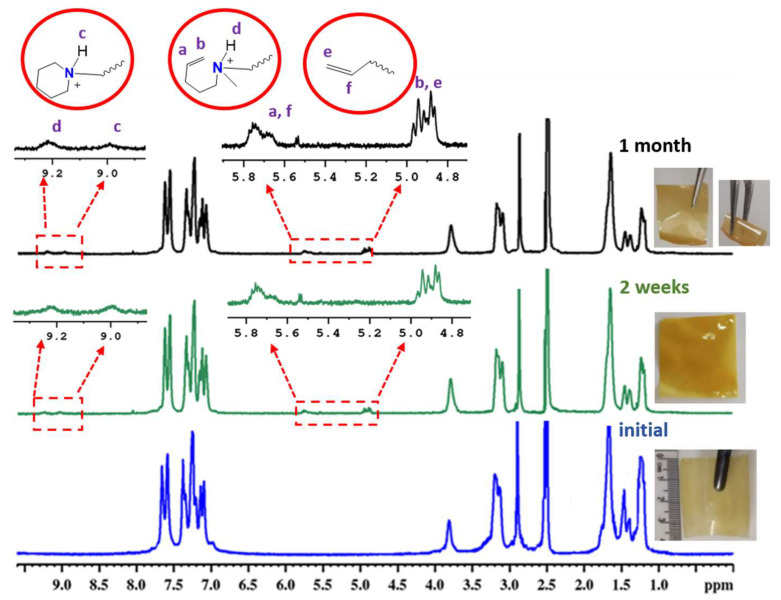
^1^H NMR spectra of piperidinium-based AEM before and after storage in 2M KOH for 1 month at 80 °C.

**Figure 10:**
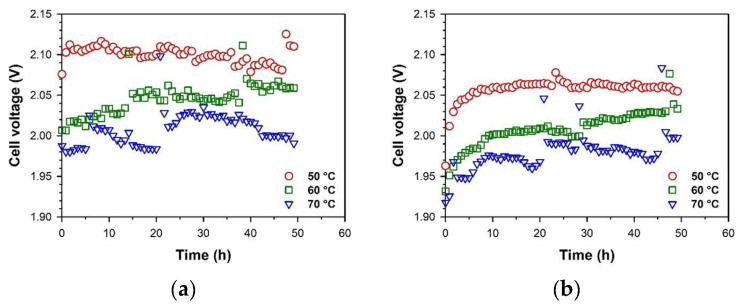
Stability test of the alkaline water electrolysis using (**a**) P(OB70pyr-TFAP) and (**b**) P(OB73pip-TFAP) membranes. Cell voltage recorded at 250 mA cm^−2^. 1M KOH, temperature indicated in the figure inset, Ni foam as anode and cathode (4 cm^2^).

On the other hand, P(OB73pip-TFAP) was still soluble in DMSO-d_6_ after 1 month of aging; however, in order to protonate any present tertiary amines formed in degradation reactions, the sample was dissolved in a mixture of DMSO-d_6_/TFA for ^1^H NMR analysis (Figure 9). Two sets of vinylic signals (small signals) emerged in the region between 4.8 ppm and 5.8 ppm after 2 weeks’ alkaline treatment, and their intensity slightly increased with increasing aging time (after 1 month). The first set of signals observed (at ~4.9 and ~5.8 ppm, corresponding to **CH_2_**= and **CH**-CH_2_, respectively) strongly implies that Hofmann β-elimination degradation of the piperidinium ring took place, while the second set of signals (at ~4.8 and ~5.7ppm, assigned to **CH_2_**= and **CH**-CH_2_, respectively) is attributed then to β-elimination in the alkyl spacer chain [27,46,49,50]. In addition, two different signals from protonated amines were detected at ∼9.2 and ∼9.0 ppm, respectively, where the former is ascribed to ring-opening β-elimination and the latter was consequently attributed to nucleophilic substitution at the methyl position of the piperidinium ring [51]. The degradation products are depicted in Figure 9. The third degradation pathway of nucleophilic methyl substitution was further confirmed by the decreased intensity of methyl protons at 2.9 ppm with respect to the aromatic protons after alkaline treatment. It can be concluded that the degradation of P(OB73pip-TFAP) already started in the first 2 weeks, while the P(OB70pyr-TFAP) membrane showed no signs of degradation over the same period. It is worth mentioning that, although the conductivity of both samples increased during the first 2 weeks of the alkaline stability test, in the case of P(OB73pip-TFAP), the ^1^H NMR data unveiled that degradation occurred via three different degradation pathways, resulting in ionic loss. In addition, the ionic loss was also confirmed by the reduction in the ratio of the signal intensity of the aliphatic protons and the signal of aromatic protons. Thus, it can be confirmed that the decrease in conductivity mainly comes from the degradation of pyrrolidinium and piperidinium groups. It should be mentioned that both membranes retained their flexibility and mechanical stability after the prolonged alkaline treatment (Figure 9 and Figure 10), underlying the robust alkaline stability of the polymeric backbone. These results suggest that the high alkaline stability of pyrrolidinium-based AEM is primarily attributed to its alkali-resistant oxindole backbone [27,32,50], the high alkaline stability of pyrrolidinium due to its low strain and large energy barrier towards E2 elimination [23] and to the steric hindrance induced by the long alkyl chain, thus protecting the cation from hydroxide attack [52,53]. In addition, the long alkyl chain provides a high electron density around the β-H, inhibiting the Hofmann elimination.

To complement the NMR and conductivity data, ATR spectroscopy was used to study the aged samples (Figure S6 and Figure S7, respectively). No new peaks were observed after alkaline treatment, and thus it could not provide more information regarding the potential degradation of AEMs. TGA analysis was employed to study the thermal decomposition of AEMs after alkaline treatment in 2M KOH at 80 °C for 1 month. As depicted in Figure S8, TGA traces of the pyrrolidinium-based AEM were quite close to the initial traces recorded before the test. In conclusion, the TGA data are in line with the results derived from ^1^H NMR analysis, and the studied membranes still have high thermal stability after the aging test.

### 3.7. AEMWE Electrolysis Testing

In order to evaluate characteristics of the prepared membranes under the current load, alkaline water electrolysis in a laboratory single-cell electrolyzer was performed in 1M KOH at temperatures of 50, 60 and 70 °C. The properties of the membranes under the current load can generally differ from those obtained ex situ, due to the presence of the OH^−^ flux through the membrane. During the alkaline water electrolysis test in this work, all components and conditions were identical for the P(OB70pyr-TFAP) and P(OB73pip-TFAP) membranes, respectively. All possible changes thus can be addressed to the changes in the membrane properties. To evaluate and compare the performance of the prepared membranes, Figure 10 shows the results of the stability test at a constant current density of 250 mA cm^−2^.

Several pieces of information can be drawn from Figure 10. Firstly, with higher temperatures, the cell voltage decreases in the case of both tested membranes. This has two reasons. The first reason relates to the increased ionic conductivity of both membranes, which is in accordance with ionic conductivity dependence on temperature, shown in Figure 6 and Figure 7. The second explanation of the improved performance at higher temperatures lies in the increased kinetics of the electrode reactions, as it follows from the Arrhenius law.

Another piece of information provided by Figure 10 follows from the cell voltage comparison for the P(OB70pyr-TFAP) and P(OB73pip-TFAP) membranes. It is clearly seen that the P(OB73pip-TFAP) membrane showed lower cell voltage, i.e., higher efficiency of the alkaline water electrolysis, compared to the P(OB70pyr-TFAP). Considering that the changes in the cell voltage are only due to differences in the membrane’s properties, this observation can be explained by the lower ohmic resistance of the P(OB73pip-TFAP) membrane. Lower ohmic resistance can then be achieved due to the higher ionic conductivity of the P(OB73pip-TFAP) membrane (see Figure 6) or its lower thickness.

However, it is evident that cell voltage dependence on time has different shapes for the P(OB70pyr-TFAP) and P(OB73pip-TFAP) membranes. In the case of the P(OB70pyr-TFAP) membrane, the cell voltage at 50 °C is decreasing within 50 h of the experiment. This decrease in the cell voltage can be connected with the polymer matrix relaxation and consequent ionic conductivity improvement. At a temperature of 60 °C, an increase in the cell voltage of about 50 mV can be observed within first 20 h of operation, followed by the cell voltage stabilization. At 70 °C, the cell voltage for the P(OB70pyr-TFAP) membrane shows fluctuations (2.01 V ± 0.02 V), but the initial and last values are the same (1.99 V).

On the other hand, in the case of P(OB73pip-TFAP), it is possible to observe a sharp cell voltage increase at the beginning of each measurement. Even more, at temperatures of 60 and 70 °C, the cell voltage is continuously increasing within the stability test. Differences in the stability curves shape resulted in the equal cell voltage values at the end of the 70 °C stability experiment (1.99 V for both membranes), despite the fact that cell voltages 2.08 V and 1.96 V have been recorded at the beginning of the stability test for the P(OB70pyr-TFAP) and P(OB73pip-TFAP) membrane, respectively. These results are supported by the load curve measurements, which were performed at the beginning and at the end of the measurement at particular temperatures (Figure S9).

**Figure 11:**
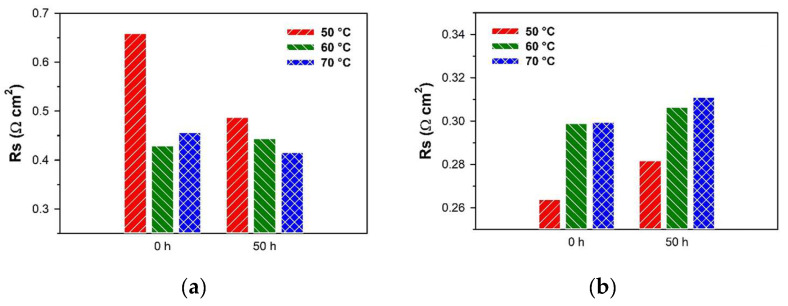
Values of laboratory single-cell electrolyzer ohmic resistance (Rs) for (**a**) P(OB70pyr-TFAP) and (**b**) P(OB73pip-TFAP) membranes at beginning (0 h) and end (50 h) of the stability test at particular temperatures. Rs evaluated from EIS measurement at 1.8 V under conditions of alkaline water electrolysis. 1M KOH, temperature indicated in the figure inset, Ni foam as anode and cathode (4 cm^2^).

Observed behavior is in accordance with the ex situ stability experiments shown in Figure 7. However, in order to further confirm the results of the alkaline water electrolysis, the values of the ohmic resistance (Rs) of the laboratory single-cell electrolyzer are shown in Figure 11. Due to the setup used and conductivities of the cell’s components, the values of Rs can be attributed to the resistance of the membrane used. Data have been collected at the beginning (0 h) and at the end (50 h) of the experiment for particular temperatures.

When compared in Figure 11a,b, it is nicely seen that the P(OB70pyr-TFAP) membrane shows higher values of Rs when compared to the P(OB73pip-TFAP) membrane. This is once again in agreement with ex situ results of ionic conductivity measurements (see Figure 6 and Figure 7) and results of the alkaline water electrolysis experiments (Figure 10 and Figure S9). However, the differences are even more pronounced in this case due to differences in the thicknesses of the membranes, which were 102 and 80 µm for P(OB70pyr-TFAP) and P(OB73pip-TFAP), respectively, and because thickness influence was not corrected for the alkaline water electrolysis experiments.

It is evident from Figure 11a that ohmic resistance of P(OB70pyr-TFAP) decreased significantly during the stability test at 50 °C. This may indicate that the membrane was further swollen under the conditions of the alkaline water electrolysis, which resulted in its higher ionic conductivity. This can be caused by the relaxation of the polymer matrix under the conditions of the alkaline water electrolysis. Such relaxation can increase the number of the accessible functional groups or can allow the formation of wider transport channels. Further decrease in the ohmic resistance was observed with increasing temperatures from 50 to 60 °C. This is common behavior for ionic conductors. During the stability test at 60 °C, a slight increase in the ohmic resistance was observed. The first possible explanation of the Rs value increase is related to the chemical degradation of the membrane. However, EIS was measured at constant cell voltage (1.8 V), which means that the current flow through the cell was higher with higher temperatures (see Figure 10 and Figure S9). Higher current means higher production of gas bubbles, which can affect the value of Rs. Another increase in the ohmic resistance was noticed with a temperature increase from 60 to 70 °C, but during the stability test at 70 °C, the value of Rs decreased. Decrease in the Rs is contrary to the assumption of the chemical degradation of the membrane.

On the other hand, for the P(OB73pip-TFAP) membrane (Figure 11b), Rs results show a significant difference between the initial (0.26 ohm cm^2^ @ 50 °C, 0 h) and final ohmic resistance (0.34 ohm cm^2^ @ 70 °C, 50 h), which is equal to 0.08 ohm cm^2^, i.e., an increase of about 31%. The first ohmic resistance increase was observed during the stability test at 50 °C. The second significant increase in ohmic resistance happened when the temperature was increased from 50 to 60 °C. Generally, the ohmic resistance was increasing gradually during the continuous measurement. The course of the Rs values for both membranes was thus significantly different. In the case of the P(OB70pyr-TFAP) membrane, Rs value showed a decreasing tendency, opposite to the case of the P(OB73pip-TFAP) membrane, where Rs value was increasing gradually.

In order to decide if the changes in the Rs values are due to the more rapid bubble formation or chemical degradation of the membrane, post-mortem analyses of IEC and ionic conductivity were performed. Post-mortem analysis showed no change of the IEC value for the P(OB70pyr-TFAP) membrane (1.45 mmol g^−1^ for fresh and after-stability sample), while the IEC of the P(OB73pip-TFAP) membrane decreased from its original value of 1.42 mmol g^−1^ to 0.71 mmol g^−1^ as evaluated after the stability test.

For the case of the ionic conductivity, the original value of the P(OB70pyr-TFAP) membrane (21 ± 1) mS cm^−1^ increased to (25 ± 6) mS cm^−1^. Increase of the ionic conductivity can be explained by the changes in the membrane structure, which facilitated ion transport and no loss of the functional groups. On the other hand, ionic conductivity of the P(OB73pip-TFAP) membrane decreased from its original value (43 ± 3) mS cm^−1^ to (36 ± 3) mS cm^−1^. A lower decrease in ionic conductivity when compared to the decrease in IEC can be explained by the adsorption of the liquid electrolyte into the bulk of the membrane. This is caused by the weaker Donnan’s exclusion (due to the reduced number of cationic groups as evidenced by the lower IEC), which allows more liquid electrolyte to be adsorbed by the membrane.

## 4. Conclusions

AEMs based on piperidinium or pyrrolidinium groups tethered to an alkaline-stable poly(oxindole biphenylene) backbone via a long flexible pentyl spacer were synthesized for use in AEM water electrolysis. Precursor copolymers were first synthesized via Friedel–Crafts polyhydroxyalkylation, followed by functionalization with long flexible alkyl chains with cationic pendants. The prepared cationic copolymers showed excellent film-forming ability, high thermal stability and displayed high electrolyte uptakes and high conductivity values at 80 °C (81.5 and 120 mS cm^−1^ for pyrrolidinium- and piperidinium-based AEM, respectively). The alkaline stability study revealed that both AEMs preserved their mechanical flexibility and robustness after one-month alkaline treatment in 2M KOH at 80 °C, although some degradation of the piperidinium-based AEM took place, as revealed by an ^1^H NMR study that agrees well with the conductivity loss observed. The results of the ex situ measurements were confirmed by the experiments carried out in the laboratory alkaline water electrolysis single-cell. Superior performance was achieved using the P(OB73pip-TFAP) membrane due to its higher ionic conductivity and lower thickness. However, this membrane showed signs of degradation even at a temperature of 50 °C. On the other hand, the P(OB70pyr-TFAP) membrane displayed lower performance, but no degradation was observed during the stability test.

## Figures and Tables

**Table 1:** Physicochemical properties of synthesized AEMs.

			Water								
Membranes	IEC (mmol g^−1^)		WU (%)		SR_a_ (%)		SR_t_ (%)		EU (%) ^a^		σ (mS cm^−1^) ^a^
	^1^HNMR	Titration	20 °C	80 °C	20 °C	80 °C	20 °C	80 °C	20 °C	80 °C	80 °C
P(OB70pyr-TFAP)	1.54	1.28	10	24	8	11	4.5	14	24	58	81.5
P(OB73pip-TFAP)	1.55	1.34	35	48	16.5	28	4.5	29	32	55	120.0

^a^ measured in 2M KOH solution.

## Data Availability

Data are contained within the article and Supplementary Materials.

## Supplementary Materials

The following supporting information can be downloaded at: https://www.mdpi.com/article/10.3390/polym16010099/s1, Figure S1: ^1^H NMR spectrum of 1-(5-bromopentyl)-1-methylpyrrolidinium bromide (BrPyr); Figure S2: ^1^H NMR spectrum of 1-(5-bromopentyl)-1-methylpiperidinium bromide (BrPip); Figure S3: ATR spectra of precursor copolymer P(OBx-TFAP) and pyrrolidinium- and piperidinium-based AEMs in bromide form; Figure S4: AFM and TEM images of pyrrolidinium- (a,b) and piperidinium- (c,d) based AEMs in bromide form; Figure S5: Water uptake and swelling behavior of (a) P(OB70pyr-TFAP) and (b) P(OB73pip-TFAP) in the OH^−^ form as a function of temperature; Figure S6: ATR spectra of pyrrolidinium-based AEM after storage in 2M KOH at 80 °C for 2 weeks and 1 month. The inset corresponds to the region from 1700 to 900 cm^−1^; Figure S7: ATR spectra of piperidinium-based AEM after storage in 2M KOH at 80 °C for 2 weeks and 1 month. The inset corresponds to the region from 1700 to 900 cm^−1^; Figure S8:TGA curves of pyrrolidinium-based AEM (hydroxide form) before and after storage in 2M KOH at 80 °C for 2 weeks and 1 month; Figure S9: Load curves measured for (A–C) P(OB70pyr-TFAP) and (D–F) P(OB73pip-TFAP). 1 mol dm^−3^ KOH at temperatures 50 (a,d), 60 (b,e) and 70 °C (c,f), Ni foam electrodes (4 cm^2^ geometrical area); Figure S10: Equivalent electrical circuit used for EIS spectra evaluation. R2—ohmic resistance; R1—polarisation resistance; R4—resistance of the pores in Ni foam.
