# Supplementary material for: Cycloaliphatic Quaternary Ammonium Functionalized Poly(oxindole biphenyl) Based Anion-Exchange Membranes for Water Electrolysis: Stability and Performance
Source: Polymers (Basel). 2023 Dec 28;16(1):99. doi: 10.3390/polym16010099 (PMC10780940; doi:10.3390/polym16010099)
Supplement: Supplementary file 1 [file polymers-16-00099-s001.zip › polymers-2791422-supplementary.pdf]

Supplementary Material

Cycloaliphatic quaternary ammonium functionalized poly (oxindole biphenyl) based Anion Exchange membranes for Water Electrolysis: Stability and Performance

Sara Gjoshi[1], Paraskevi Loukopoulou[1], Michaela Plevova[2], Jaromir Hnat[2], Karel Bouzek[2], Valadoula Deimede*[1]

[1]*Department of Chemistry, University of Patras, GR-26504, Patras, Greece*

[2]*University of Chemistry and Technology, Prague, Department of Inorganic Technology, Technická 5, Prague 6, Czech Republic*

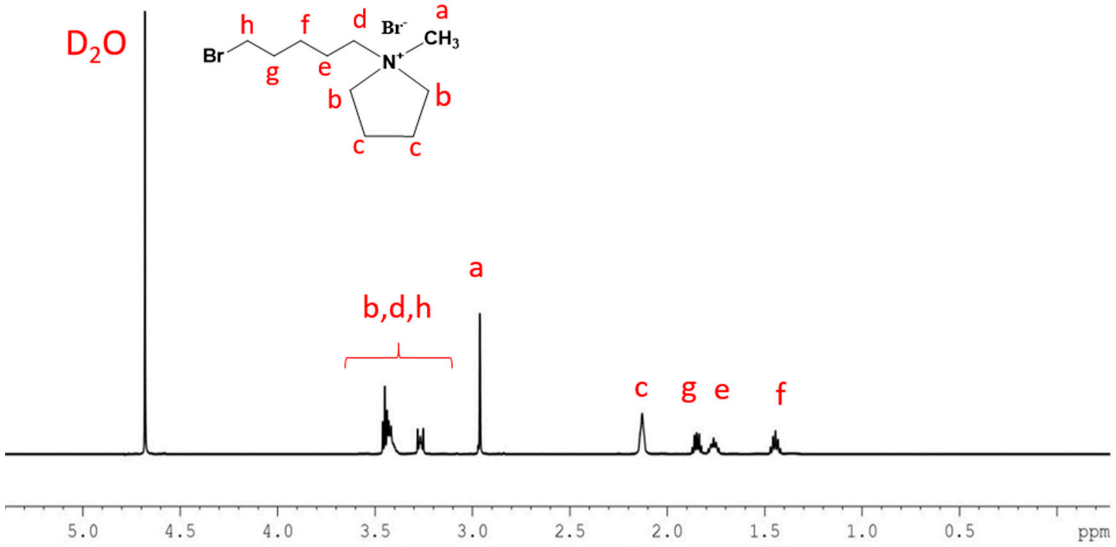

Figure S1. ^{1}H NMR spectrum of 1-(5-bromopentyl)-1-methylpyrrolidinium bromide (BrPyr).

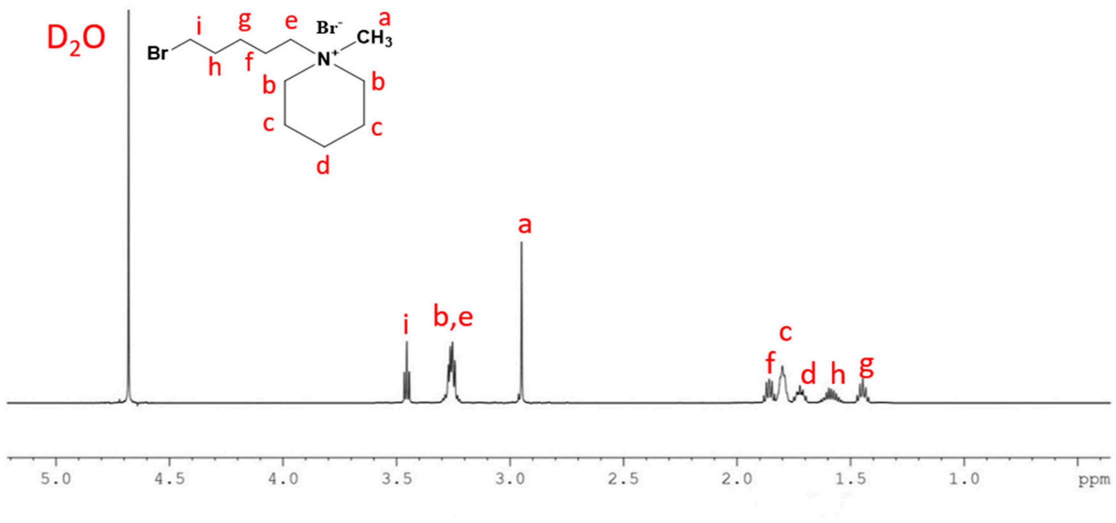

Figure S2. ^{1}HNMR spectrum of 1-(5-bromopentyl)-1-methylpiperidinium bromide (BrPip).

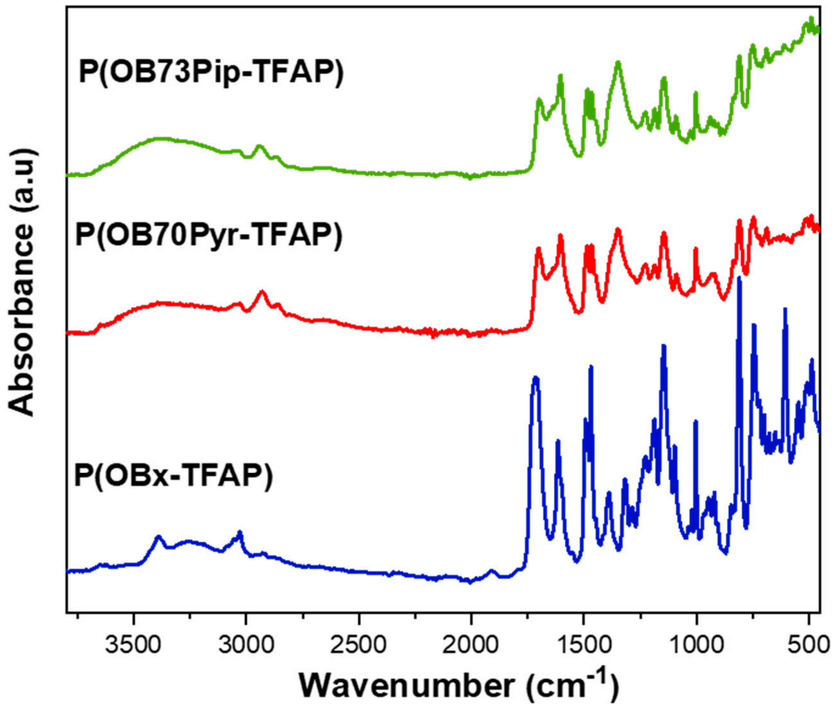

Figure S3. ATR spectra of precursor copolymer P(OBx-TFAP) and pyrrolidinium and piperidinium based AEMs in bromide form.

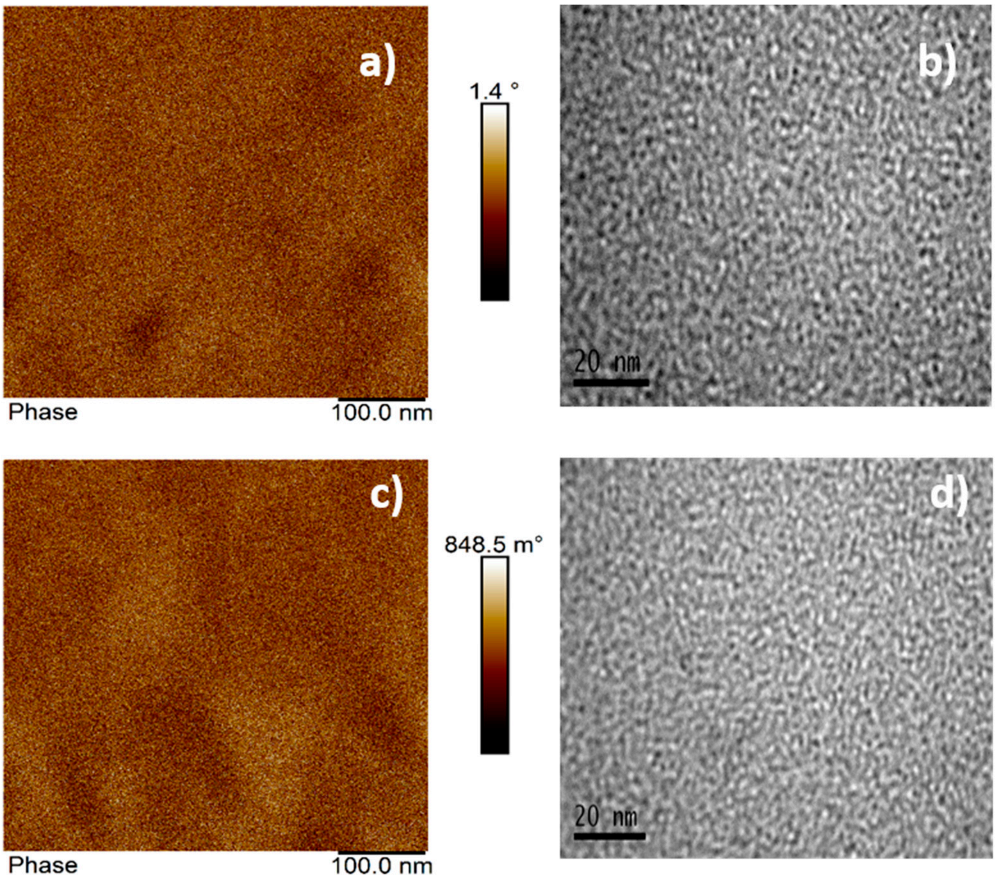

Figure S4. AFM and TEM images of pyrrolidinium (a and b) and piperidinium (c and d) based AEMs in bromide form.

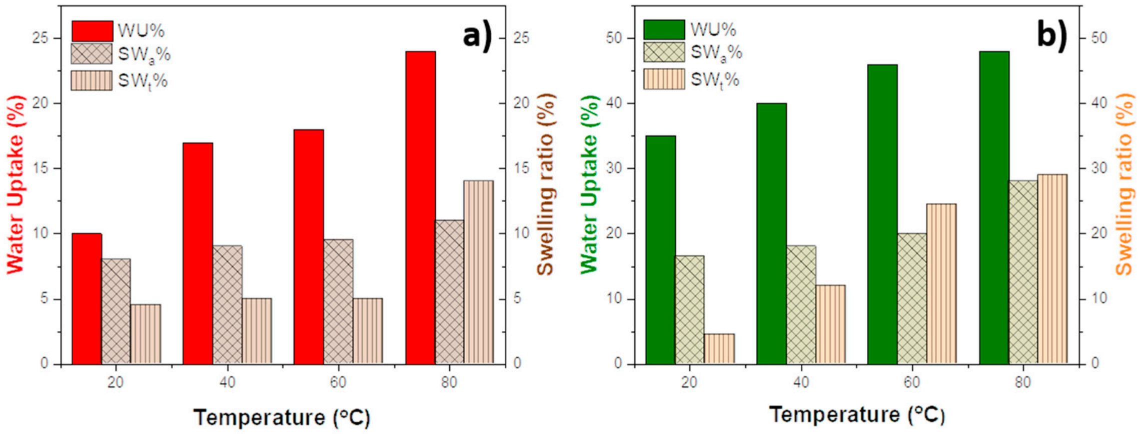

Figure S5. Water uptake and swelling behavior of a) P(OB70pyr-TFAP) and b) P(OB73pip-TFAP) in the OH^- form as a function of temperature.

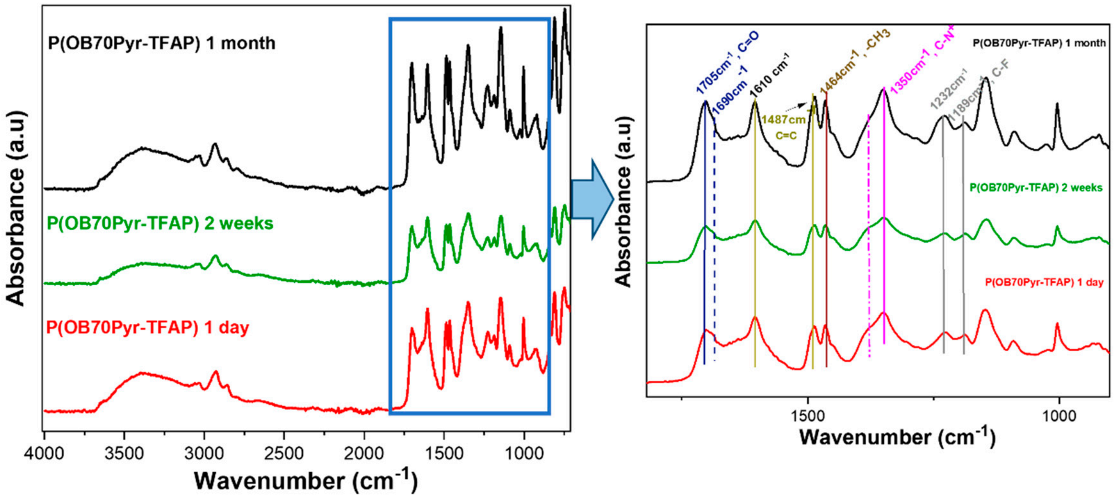

Figure S6. ATR spectra of pyrrolidinium based AEM after storage in 2M KOH at 80 C for 2 weeks and 1 month. The inset corresponds to the region from 1700 to 900 cm^{-1}.

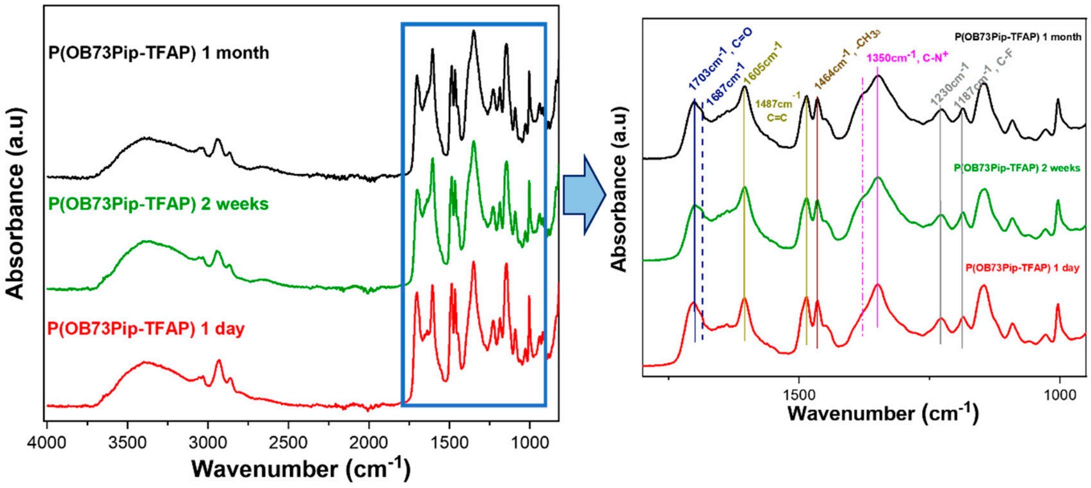

Figure S7. ATR spectra of piperidinium based AEM after storage in 2M KOH at 80 °C for 2 weeks and 1 month. The inset corresponds to the region from 1700 to 900 cm^{-1}.

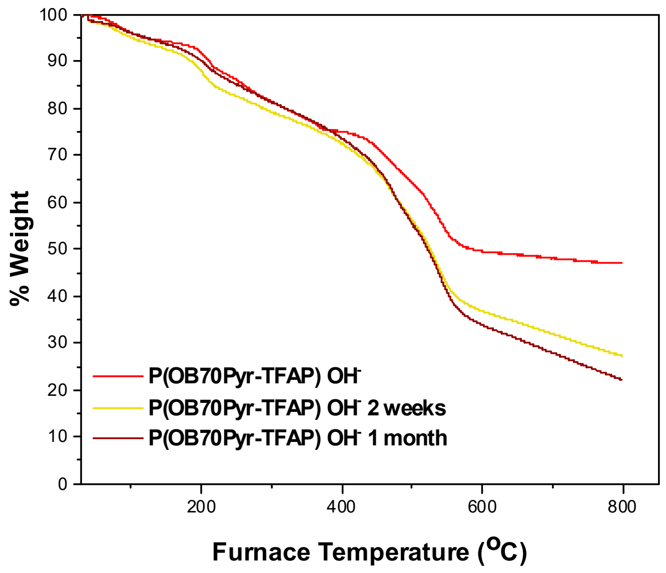

Figure S8. TGA curves of pyrrolidinium based AEM (hydroxide form) before and after storage in 2M KOH at 80 °C for 2 weeks and 1 month.

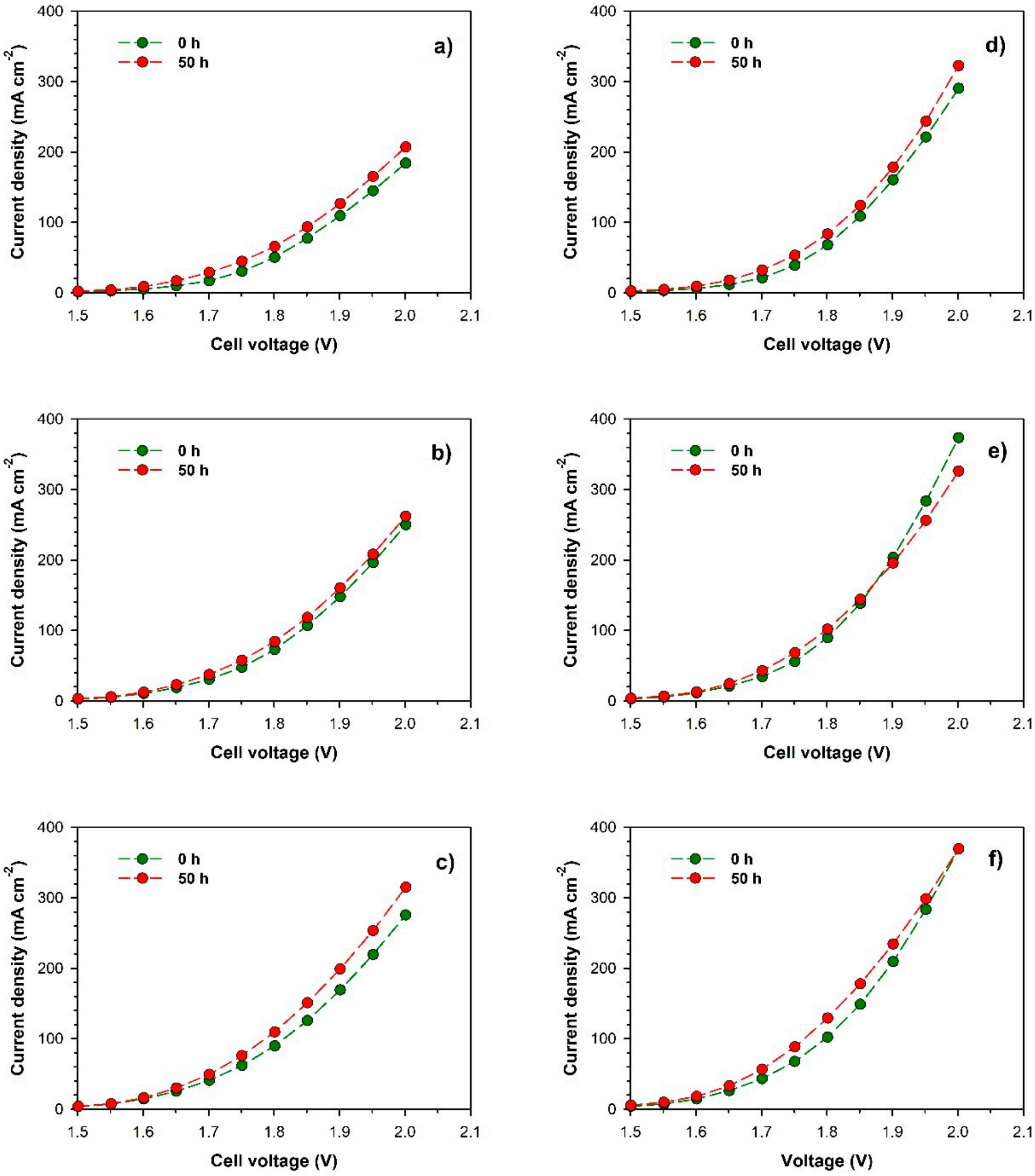

Figure S9. Load curves measured for (A–C) P(OB70pyr-TFAP) and (D–F) P(OB73pip-TFAP). 1 mol dm^{-3} KOH at temperatures 50 (a, d), 60 (b, e) and 70 °C (c, f); Ni foam electrodes (4 cm^2 geometrical area)

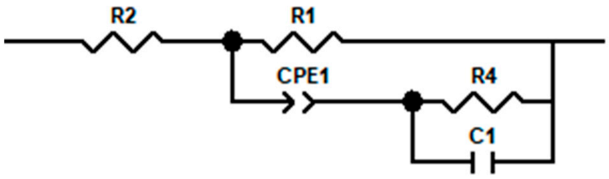

Figure S10. Equivalent electrical circuit used for EIS spectra evaluation. R2 – ohmic resistance; R1 – polarisation resistance; R4 – resistance of the pores in Ni foam
